# Alcohol consumption and risk of uterine myoma: A systematic review and meta analysis

**DOI:** 10.1371/journal.pone.0188355

**Published:** 2017-11-27

**Authors:** Francesca Chiaffarino, Sonia Cipriani, Elena Ricci, Carlo La Vecchia, Vito Chiantera, Alessandro Bulfoni, Fabio Parazzini

**Affiliations:** 1 Dipartimento della Donna, del Neonato e del Bambino, Fondazione IRCCS Cà Granda, Ospedale Maggiore Policlinico, Milan, Italy; 2 Dipartimento di Scienze Cliniche e di Comunità, Università degli studi di Milano, Milan, Italy; 3 Department of Gynecology, Charitè Universitätsmedizin, Berlin, Germany; 4 Unità di Ostetricia e Ginecologia, Humanitas San Pio X Hospital, Milan, Italy; Erasmus MC, NETHERLANDS

## Abstract

**Background:**

The published data about alcohol consumption and uterine myoma are scanty and controversial: some studies found positive association whereas other studies showed no association.

**Objectives:**

To conduct a systematic review and meta-analysis to determine whether alcohol is a risk factor for myoma.

**Search strategy:**

A MEDLINE/EMBASE search was carried out, supplemented by manual searches of bibliographies of the selected studies.

**Selection criteria:**

Articles published as full-length papers in English. In the review we included all identified studies. Otherwise, the inclusion criteria for studies included in the meta-analysis were: a) case-control or cohort studies, reporting original data; b) studies reporting original data on the association between alcohol consumption and myoma; c) diagnosis of myoma was ultrasound or histological confirmed and/or clinically based.

**Data collection and analysis:**

A total of 6 studies were identified for the review and 5 studies were included in the meta-analysis. The primary outcome was the incidence of uterine myoma in ever versus never alcohol drinkers and when data were available, we also analyzed categories of alcohol intake. We assessed the outcomes in the overall population and then we performed a subgroup analysis according to study design. Pooled estimates of the odds ratios (OR) with 95% confidence interval (CI) were calculated using random effects models.

**Main results:**

The summary OR (95%CI) of myoma forever versus never alcohol intake was 1.12 (0.94–1.34) with significant heterogeneity. The summary OR for current versus never drinking was 1.33 (1.01–1.76) with no heterogeneity.

**Conclusions:**

Ever alcohol consumption is not associated with myoma risk. Based on the data of two studies, current alcohol drinkers had a slightly borderline increased risk of diagnosis of myoma. In consideration of the very limited number of studies and the suggestion of a potential increased risk among current drinkers, further studies are required.

## Introduction

Uterine myoma, a benign smooth muscle tumor, is the most common tumor of the female reproductive tract. The incidence and prevalence of myomas remain currently unknown. Available data are difficult to compare due to differences in the study population and screening methods of the published studies. Taking into consideration the above observations, prevalence data range from 5% to 21% [1–3].

Many different risk factors have been associated with myoma occurrence; age, race, reproductive and lifestyle factors are included among them, but their role is still not well defined nor fully understood [4–7]. It has been established that incidence rates of myoma are higher among Black women, but the biologic basis is unclear [5]. Birth and Depo-Provera (a contraceptive method for women) use has been shown to decrease myoma risk [4, 8]; likewise, a diet rich in vegetables, fruit and soya food has been associated with decreased frequency of myoma [7, 9].

There is consensus that uterine myoma is an estrogen-dependent condition [10]; any factor that reduces endogenous estrogen levels and increases progesterone levels may reduce the risk of uterine myoma.

Alcohol consumption is a common habit worldwide and is associated with higher endogenous levels of estradiol and estrone [11–13]. Alcohol promotes aromatase activity, increasing estrogen levels. Alcohol could also interact with luteinizing hormone production from the pituitary gland, increasing estradiol release from the ovaries [14]. Long-term alcohol intake may also affect immune system and may regulate production of pro-inflammatory cytokines [15].

According to the available evidence, the relation between alcohol consumption and uterine myoma risk is controversial. Prospective cohort studies found a positive association with current alcohol consumption [5, 6], but no association emerged in two case control studies [7, 16] and there are no systematic reviews on this issue. The results of a systematic review may be important for health authorities: identifying modifiable risk factors, like alcohol consumption, could be decisive for the prevention of these tumors. To offer a general figure of the available data on the potential relation between alcohol and myoma, and to provide an overall quantitative estimate of any such relation, we conducted a systematic review and a meta-analysis of studies published up to May 2017.

## Materials and methods

### Data source and search strategy

We performed a MEDLINE/EMBASE search of papers published until May, 2017, using the Medical Subject Heading terms “uterine myoma” or “leiomyoma” combined with “alcoholic beverages" or "alcohol drinking" or "vegetables" or "fats" or “vitamins" or "diet" and free search terms “alcohol” or “alcoholic beverages”or “alcohol consumption”or “alcohol drinking”or “wine” or “beer” or “diet” or “vegetable” or “nutrition” or “vitamin” or “fat” in combination with “fibroids” or “uterine fibroids” or “myoma” or “uterine myoma” or “leiomyoma” or “uterine leiomyoma” (S1 File). The meta-analysis was reported following the MOOSE (Meta-analysis of Observational Studies in Epidemiology) [17] and PRISMA (preferred reporting items for systematic reviews and meta-analyses) [18] guidelines (S1 Table).

### Study selection and eligibility criteria

We selected only studies on humans, published as full-length papers in English. Moreover, bibliographies of the retrieved papers were reviewed, to identify any other relevant publication.

Studies were included if they fulfilled the following criteria: a) case-control or cohort studies, reporting original data; b) studies reporting information on the association between alcohol consumption and myoma, and/or estimates of the relative risk (RR) or the odds ratio (OR), with the corresponding 95% confidence intervals (CI), or frequency distribution to calculate them; c) studies where diagnosis of myoma was ultrasound or histological confirmed and/or clinically based. The exclusion criteria were: a) cross-sectional studies, since in this study design exposure and disease are recorded at the same time and it could not determine whether the exposure preceded the occurrence of uterine myoma, unless the studies included only newly-diagnosed myoma cases; b) studies where myoma diagnosis was exclusively self-reported.

When we found more than one publication based on the same study population, we included only the one with most detailed information, or published most recently.

### Data extraction and quality assessment

Data extraction and selection of eligible studies were carried out in duplicate by two researchers (FC and SC). Disagreements were solved by discussing and reviewing the different respective issue that emerged. Cross-referencing of selected articles revealed no further eligible records.

From each publication we extracted the following information: country of origin; study design; number and characteristics of subjects (cases, controls or cohort size); age, if available; categories of alcohol intake; measures of association (RR or OR) of myoma and corresponding 95% CI for every category of alcohol intake, or frequency distribution to calculate them; confounding variables allowed for in the statistical analysis. When more than one regression model was provided, estimates adjusted for the largest number of confounding variables were considered.

The quality of the studies included in the review was assessed using the Newcastle-Ottawa scale. This instrument was developed to assess the quality of nonrandomized studies, specifically cohort and case–control studies [19]. Studies were judged based on three broad categories: selection of study groups, comparability of study groups, and assessment of outcome (cohort studies) or ascertainment of exposure (case-control studies). The maximum score was 9.

### Data synthesis and analysis

Pooled estimates of the OR and the corresponding 95% CI were calculated using random effects models.

We assessed the heterogeneity among studies using the χ^2^ test [20] and quantified it using the I^2^ statistic. Results were defined as heterogeneous for P values less than 0.10. The funnel plot and Egger’s test [21] were used to detect publication bias

We computed summary estimates for ever and current alcohol consumption, as compared to never alcohol consumption.

## Results

**Fig 1:**
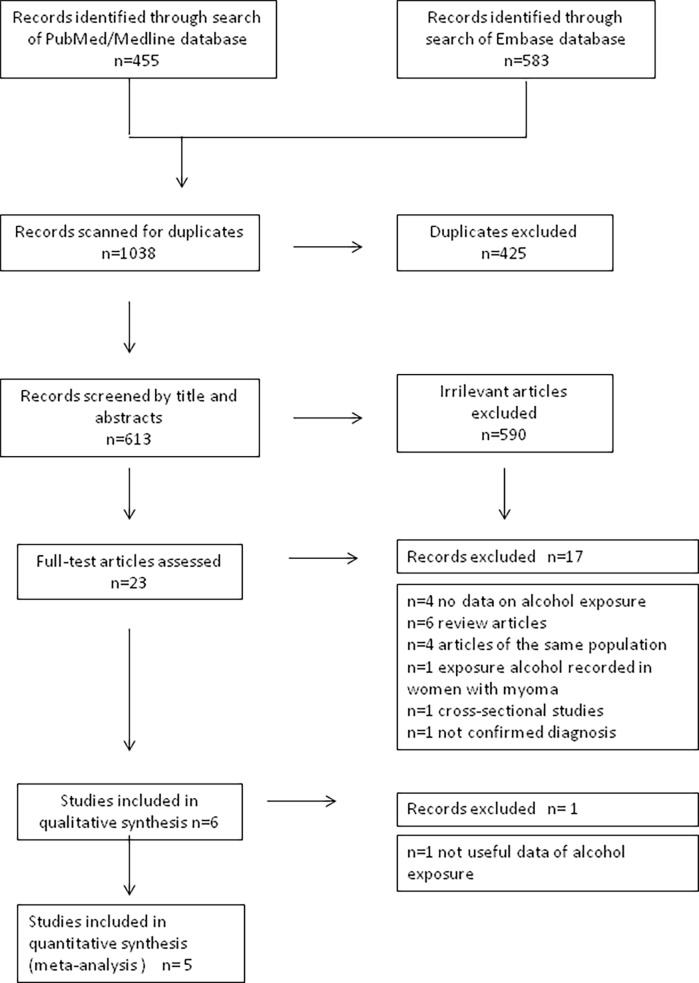
Flow chart of the selection of studies on alcohol intake and risk of uterine myoma included in the systematic review and meta-analysis.

Fig 1 shows the flow-chart of the selection of publication. In total, after deduplication, we identified 613 potentially relevant citations. Based on the title and abstracts, full texts of 23 articles were selected for detailed evaluation. Of these articles, 17 articles were excluded for reasons shown in Fig 1. The remaining 6 articles met our eligibility criteria and were therefore included in the analysis.

### Summary of included studies

A total of 6 studies reporting data on 486068 women with 9315 myoma were included in this review. Table 1 shows the main characteristics of the selected studies: four cohort, of which one was retrospective and two case-control studies. Of these, three studies were from USA [5, 6, 22], 1 from Asia [16] and 2 from Italy [7, 23]. The articles were published between 1997 and 2013. Media follow-up time of cohort studies was 6.33 years (range 4–11). The quality of the studies was assessed using the Newcastle-Ottawa Scale where 9 was the maximum score. The selected studies scored from 6 to 8. Two studies scored 6, one 7 and three studies scored 8.

**Table 1:** Main characteristics of the selected studies on alcohol consumption and risk of uterine myoma.

Study and year	Country	Study design	Cases	Controls	Sample size cases/ controls	Age (ys)	Alcohol habits	Confounding factors	NOS Quality score
Chiaffarino et al., 1999	Italy	Case-control	Women with myoma (histologically confirmed)	Women admitted to the hospitals for acute, non-gynecologic, nonhormonal, nonneoplastic conditions	843/1557	Median age 43. Range 21–54	Total daily average alcohol intake (tertiles)	Age, education, marital status, menopausal status, BMI, parity, smoking	7
Eskenazi et al., 2007	Italy	Retrospective cohort	Women with myoma (medical record or ultrasound confirmed)	Women without myoma (ultrasound screening)	251 / 956	Mean age 40.4 (standard deviation 11.5)	Never, former current drinkers		8
He et al., 2013	China	Case-control	Women with myoma (surgically or ultrasound confirmed)	Women without myoma (ultrasound screening)	73 / 210	73% of cases and 75% of controls: < 56 years	Never, ever		6
Marshall et al., 1997 ^a^	USA	Cohort	Women with myoma (ultrasound/hysterectomy confirmed)	Women without myoma	4181 incident cases among 327065 premenopausal women	25–42	Mean current alcohol intake (g/d)		
Templeman et al., 2009	USA	Cohort	Women with myoma (surgically confirmed)	Women without myoma	1790 cases among 133,000 women	25–84	None, < 20 g, ≥20 g/day	Race, family history of fibroids, BMI, menopausal hormone status, age at first full-term pregnancy	6
Wise et al., 2004	USA	Cohort	Women with myoma (ultrasound or hysterectomy confirmed)	Women without myoma	2177 incident cases among 21885 premenopausal women	Only premenopausal women	Never, former and current drinkers	Age, time period, age at menarche, parity, age at first and last birth, education, BMI, OC use, smoking, caffeine intake.	8

^a^This study did not contribute in the meta-analysis

### Alcohol consumption and myoma risk

The American cohort study of Marshall et al. [5] was not included in the meta-analysis because the paper summarized the finding but did not provide the estimates on the association between alcohol consumption and myoma risk. The study reported, in premenopausal nurses, positive association of incidence of myoma with current alcohol consumption. Therefore, the data from the other 5 studies, based on 159003 women and reporting 5134 myoma cases contributed to the meta-analysis.

The Black Women’s Health prospective cohort study found that myoma risk was positively associated with duration of alcohol consumption (20 years or more) and current alcohol intake, particularly beer (7 or more drinks per week) [6]. Similarly, in California Teachers cohort study, drinking 20 g or more of alcohol per day was associated with surgery for myoma [22]. In this study the researchers assigned a serving of beer to 13.2 g of alcohol, a glass of wine to 11.1 g of alcohol and a shot of liquor to 15.0 g of alcohol. The Black Women’s Health Study analyzed separately the role of different types of alcoholic beverages on myoma risk: it showed a stronger association for beer consumption than for wine or liquor consumption [6].

The two case-control studies reported no association between myoma and alcohol consumption [7, 16].

**Fig 2:**
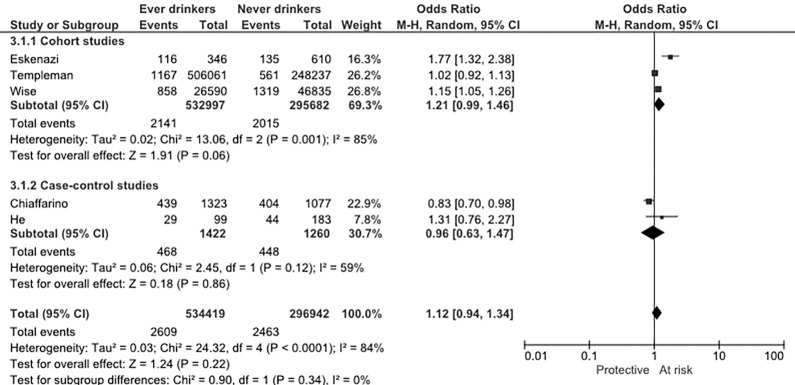
Study specific and summary odds ratio (OR) of uterine myoma for ever versus no alcohol intake. CI: confidence interval.

Fig 2 shows the study-specific and pooled OR for ever versus never alcohol intake. Not all studies presented estimate of ever alcohol consumption category versus never, thus we summarized data to obtain ever alcohol intake category. The summary OR (95%CI) of myoma for ever alcohol intake was 1.12 (0.94–1.34) with significant heterogeneity. The pre-planned subgroup analysis by study design was performed: the OR in case-control studies was 0.96 (95% CI: 0.63–1.47) and in cohort studies was 1.21 (95%CI: 0.99–1.46), neither of them was statistically significant.

**Fig 3:**
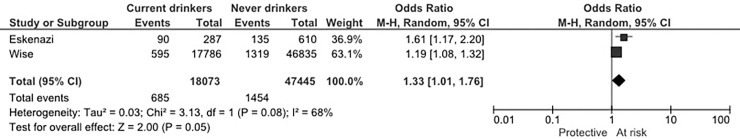
Study specific and summary odds ratio (OR) of uterine myoma for current versus no alcohol intake. CI: confidence interval.

Only two cohort studies (one of these retrospective) reported estimates for current and former versus never alcohol consumption. Fig 3 showed the summary OR of current versus never drinkers, 1.33 (95% CI: 1.01–1.76) with no heterogeneity. The summary OR of former versus never drinkers was 1.65 (95% CI: 0.65–4.22), not statistically significant and with significant heterogeneity (data not shown).

We did not find any relevant asymmetry in the funnel plot (S1 Fig), and the Egger’s test was not statistically significant (p = 0.699).

## Discussion

Findings from this systematic review and meta-analysis are based on a very limited number of studies, thus the results should be considered cautiously. Taking this aspect into consideration, the general results suggested that ever alcohol consumption is not associated with myoma risk. Subgroup analysis for study design showed, among cohort studies, a modest increased risk of ever alcohol drinkers on myoma risk, but this result was not statistically significant.

Based on the data of two studies, current alcohol drinkers had a slightly borderline increased risk of diagnosis of myoma.

This systematic review was conducted in order to clarify the potential association between alcohol and uterine myoma. As alcohol consumption is a modifiable risk factor, this association could have clinical interest and health policy consequences.

This review and meta-analysis may be affected by potential limitations. In the selected studies, women’s ethnicity was heterogeneous: it has been observed a higher prevalence of myoma in Black women compared with White women, but the excess rates were not attributable to a higher prevalence of risk factors among Black women [5].

Moreover, there is a difference in the women population enrolled in the studies: the California Teachers Study included perimenopausal and postmenopausal women who were not enrolled in previous cohort studies [5, 6], but the proportion of women aged more than 50 was relatively low [22].

The studies included in the meta-analysis showed difference in the ascertainment of the myoma absence in the control group: in some studies the women were ultrasound screened, in other the absence of myoma was self-reported and we cannot exclude the presence of misclassified women with asymptomatic or otherwise undiagnosed myoma in the control group, resulting in a potential underestimation of the reported effect. Thus, these papers could be looking at whether alcohol increases the risk of women having symptomatic or clinically recognized myoma or severe cases of myoma (surgery required).

Moreover, in all the studies information regarding alcohol use was self-reported, thus some misclassification may have occurred. More in general alcohol drinking, and in particular heavy drinking, may be misreported in observational studies [24, 25]. However, studies investigating reproducibility and validity of self-reported alcohol drinking in various populations found satisfactory correlation coefficients [26–28]. In general, however, any misclassification should tend to reduce the odds ratio estimates.

The only study that presented the effect of duration of alcohol intake showed a positive association with years of alcohol consumption [6]. Likewise, this study analyzed separately the effect of different alcoholic beverages, showing stronger positive association for beer intake than for wine or liquor consumption. It could be that beer exerts a different effect than other types of alcoholic beverages on hormone-dependent disease, like myoma. No other studies were able to analyze separately the effect of different alcoholic beverages; this fact could explain the lack of association between alcohol and myoma in some studies.

The analysis of the two studies that have presented the data for current drinkers has suggested a slightly increased myoma risk. Likewise, the Nurses’ Health Study II cohort of premenopausal women showed current alcohol consumption positively associated with myoma risk, but we couldn’t include in our meta-analysis because alcohol intake original data were not reported [5]. Moreover, this finding was also confirmed in a published scientific abstract based on the cross-sectional Uterine Fibroid Study, where current alcohol consumption increased myoma risk [29].

The underlying mechanisms linking alcohol intake and myoma risk are unknown. Alcohol consumption can cause hormonal changes which could play an important role in myoma development. Alcohol intake is able to decrease estrogen metabolism, thereby increasing levels of endogenous estrogen [11–13]. Along this line, alcohol consumption is found to delay menopause onset [30]. Alcohol may interact with luteinizing hormone regulating estradiol release from the ovaries and estrogen is believed to promote the growth of myoma [31]. Thus, high levels of estrogen, as well as, growth factors, such as insulin-like growth factor (IGF) and epidermal growth factor (EGF), may promote growth of myoma [32, 33], thus current drinkers may be at increased risk.

As regards another benign estrogen-dependent disease, our recent meta-analysis provides evidence for a significant positive association between alcohol consumption and endometriosis risk [34]. Moreover, alcohol has been consistently found to increase the risk of developing estrogen-dependent diseases such as breast cancer [35]. However, several studies showed no association with endometrial cancer [36–38], which raises the questions about estrogenic effects being the primary mechanism.

The results of a systematic review may be important for health authorities: identifying modifiable risk factors, like alcohol consumption, could be decisive for the prevention of these tumors. If further epidemiological studies confirm an increased risk among current drinkers, limiting alcohol intake might be an effective public health measure in order to help the myomas prevention.

In conclusion, the general results of this analysis suggest that ever alcohol intake is not associated to myoma risk, but in consideration of the paucity of data and the suggestion of a potential increased risk among current drinkers, further studies are required.

## Supporting information

S1 File Queries Embase/Medline_May2017. (XLSX)

S1 Table PRISMA 2009 checklist. (PDF)

S1 Fig Funnel plot. (TIFF)
