# Supplementary figures and images for: Alcohol consumption and risk of uterine myoma: A systematic review and meta analysis
Source: PLoS One. 2017 Nov 27;12(11):e0188355. doi: 10.1371/journal.pone.0188355 (PMC5703463; doi:10.1371/journal.pone.0188355)

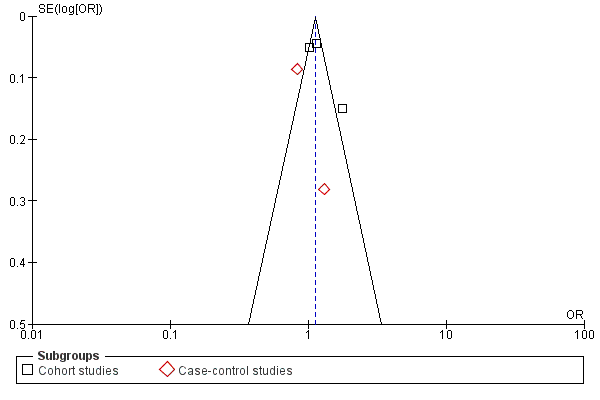

Supplement: S1 Fig — (TIFF) [file pone.0188355.s003.tiff]
